# Ventral Telencephalic Patterning Protocols for Induced Pluripotent Stem Cells

**DOI:** 10.3389/fcell.2021.716249

**Published:** 2021-08-18

**Authors:** Victor Krajka, Maximilian Naujock, Martje G. Pauly, Felix Stengel, Britta Meier, Nancy Stanslowsky, Christine Klein, Philip Seibler, Florian Wegner, Philipp Capetian

**Affiliations:** ^1^Institute of Neurogenetics, University of Lübeck, Lübeck, Germany; ^2^Department of Neurology, Hannover Medical School, Hanover, Germany; ^3^Department of Neurology, University Hospital Würzburg, Würzburg, Germany

**Keywords:** induced pluripotent stem cells, medial ganglionic eminence, Sonic hedgehog, XAV-939, purmorphamine, basal forebrain cholinergic neurons, GABAergic neurons, electrophysiology

## Abstract

The differentiation of human induced pluripotent stem cells (hiPSCs) into specific cell types for disease modeling and restorative therapies is a key research agenda and offers the possibility to obtain patient-specific cells of interest for a wide range of diseases. Basal forebrain cholinergic neurons (BFCNs) play a particular role in the pathophysiology of Alzheimer’s dementia and isolated dystonias. In this work, various directed differentiation protocols based on monolayer neural induction were tested for their effectiveness in promoting a ventral telencephalic phenotype and generating BFCN. Ventralizing factors [i.e., purmorphamine and Sonic hedgehog (SHH)] were applied at different time points, time intervals, and concentrations. In addition, caudal identity was prevented by the use of a small molecule XAV-939 that inhibits the Wnt-pathway. After patterning, gene expression profiles were analyzed by quantitative PCR (qPCR). Rostro-ventral patterning is most effective when initiated simultaneously with neural induction. The most promising combination of patterning factors was 0.5 μM of purmorphamine and 1 μM of XAV-939, which induces the highest expression of transcription factors specific for the medial ganglionic eminence, the source of GABAergic inter- and cholinergic neurons in the telencephalon. Upon maturation of cells, the immune phenotype, as well as electrophysiological properties were investigated showing the presence of marker proteins specific for BFCN (choline acetyltransferase, ISL1, p75, and NKX2.1) and GABAergic neurons. Moreover, a considerable fraction of measured cells displayed mature electrophysiological properties. Synaptic boutons containing the vesicular acetylcholine transporter (VACHT) could be observed in the vicinity of the cells. This work will help to generate basal forebrain interneurons from hiPSCs, providing a promising platform for modeling neurological diseases, such as Alzheimer’s disease or Dystonia.

## Introduction

To gain a profound understanding of diseases at the molecular level, cell models are employed for research. However, the particular use of species-specific primary human cells is severely limited by either ethical concerns, e.g., embryonic stem cell-derived samples, or the poor accessibility and low cell yield of human tissue specimens from brain surgeries (Sterneckert et al., 2014). These issues can be circumvented by using human induced pluripotent stem cells (hiPSCs), which can be differentiated into all three germ layers. For this approach, somatic cells are obtained from patients and reprogrammed into hiPSCs by the induced expression of four transcription factors (OCT4, SOX2, KLF4, and c-MYC) (Takahashi et al., 2007). The next relevant step is the development of reliable directed differentiation protocols to generate the desired cells in sufficient quantity, physiological functionality, and maturity.

**FIGURE 1:**
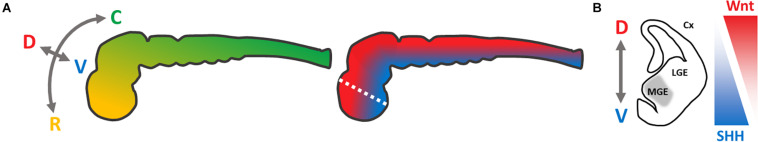
Schematic presentation of neural axes during neurogenesis of the central nervous system, modified from Petros et al. (2011). **(A)** Color-coded visualization of the rostro-caudal (yellow–green) and dorso-ventral (red–blue) axes of the neural tube; C, caudal; D, dorsal; R, rostral; V, ventral. **(B)** Coronal section of the left hemisphere (white dotted line in **A**) of the telencephalon, to visualize the targeted regional identity of the medial ganglionic eminence (gray). SHH-/Wnt-pathway activity is indicated in triangles. Cx, cortex; LGE, lateral ganglionic eminence; MGE, medial ganglionic eminence.

To model neurological diseases causing, e.g., dementia or movement disorders, the corresponding affected neural cell types can be generated using the hiPSC technology. For this purpose, hiPSCs are differentiated into neural progenitor cells (NPCs) by inducing a neuroectodermal cell fate (Chambers et al., 2009). Further differentiation of neural stem cells, without the addition of morphogens, reveals cell populations resembling the cerebral cortex (Gaspard et al., 2008). Therefore, neural differentiation into a dorsal telencephalic identity is denoted as an intrinsic “default” pathway (van den Ameele et al., 2014). To obtain specified regional identities along the rostro-caudal and dorso-ventral neural axis (Figure 1A), signaling peptides (so-called morphogens) or their small-molecule counterparts need to be administered (Petros et al., 2011). By their addition, NPCs can be patterned into desired neuronal lineages, by mimicking the neurogenesis of specific brain regions, resulting in dopaminergic, glutamatergic, and GABAergic neurons (Wang et al., 2015; Sun et al., 2016; Cao et al., 2017). For our study, we focused on the most ventral part of the telencephalon – the medial ganglionic eminence (MGE) (Figure 1B) – from which basal forebrain cholinergic neurons (BFCNs) and GABAergic interneurons arise (Sussel et al., 1999). Alterations in these cell types are associated with sporadic Alzheimer’s disease (Whitehouse et al., 1982; Coyle et al., 1983) and isolated dystonia (Levy and Hallett, 2002).

To recapitulate the neurogenesis of the MGE *in vitro*, activation of the Sonic hedgehog (SHH) pathway is required, which has a strong ventralizing impact during neural tube development (Briscoe et al., 1999; Figure 1). *In vivo* as well as *in vitro*, this leads to an upregulation of the Homeobox Protein Nkx-2.1, a key regulator for the MGE development (Ericson et al., 1996; Sussel et al., 1999; Harfe et al., 2004; Gulacsi and Anderson, 2006; Liu et al., 2013b). Alternatively, the Wnt signaling pathway can be inhibited, limiting a dorso-caudal regionalization (Backman et al., 2005). However, MGE-like cells can be generated either by solely activating the SHH pathway (Li et al., 2009; Liu et al., 2013b) or by modulating the SHH- and Wnt-pathway (Li et al., 2009; Liu et al., 2013b; Maroof et al., 2013), respectively. This combined approach seems to be more efficient.

For rostro-ventralization, the recombinant proteins SHH/DKK1 (Dickkopf-related protein 1; Wnt antagonist) can be applied (Li et al., 2009). SHH can be substituted by the small molecule purmorphamine (Smoothened activator), DKK1 by XAV-939 (tankyrase inhibitor) (Sinha and Chen, 2006; Huang et al., 2009; Liu et al., 2013a; Maroof et al., 2013; Nicholas et al., 2013). In contrast to the recombinant proteins, the latter small molecules are more cost-effective and have longer half-lives in cell culture. The concentration, timing, and duration of purmorphamine/SHH administration have a critical impact on which neuronal cell types accumulate in the *in vitro* population. These range from hypothalamic and striatal projection neurons to cortical and striatal interneurons (van den Ameele et al., 2014).

Here, we aimed to establish a protocol for the efficient generation of BFCNs and GABAergic interneurons. For this, we compared the efficiency of recombinant SHH with the small molecule purmorphamine, the most potent morphogen concentration for the upregulation of MGE-specific marker genes, and the most effective ventralization time interval. Finally, we investigated whether the neuronal cells retained their regional identity after final maturation, which resembled cholinergic neurons, and assessed their neurophysiological characteristics.

## Results

### Differentiation of Ventral Telencephalic Cell Populations From hiPSC

**FIGURE 2:**
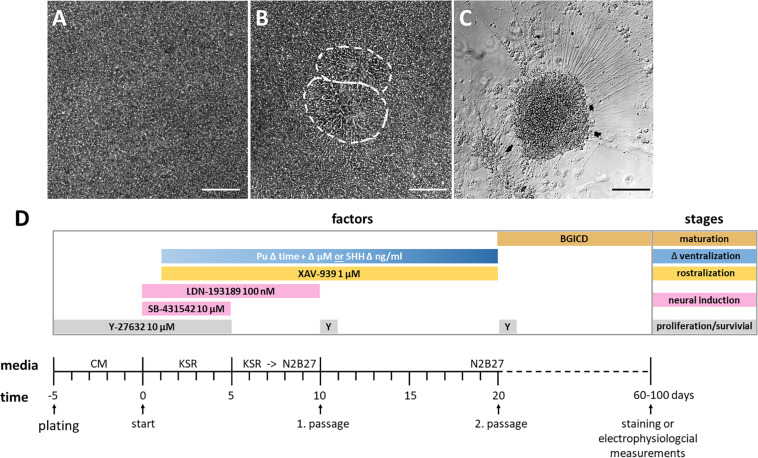
Cell morphology of each differentiation stage and schematic overview of the protocol. **(A)** Highly confluent layer of hiPSCs before neural induction, day 0. **(B)** Inner structure resembling two neural rosettes at day 13, outlined in white. **(C)** Neuronal cell cluster from which neural projections arise, day 52. All scale bars are 50 μm. **(D)** Differentiation protocol with time and factor specifications. The initiation of neural induction defines the beginning of the differentiation (day 0). The differentiation stages and utilized factors are color-coded. BGICD is an acronym of: BDNF, brain-derived neurotrophic factor; GDNF, glial cell line-derived neurotrophic factor; IGF1, insulin-like growth factor 1; cAMP, cyclic adenosine monophosphate and DAPT (Notch-pathway inhibitor); CM, MEF-conditioned hiPSC medium; KSR, knockout serum replacement medium; Pu, purmorphamine; SHH, Sonic hedgehog; Y, Y-27632 [Rho-associated protein kinase (ROCK) inhibitor, for increased cell survival].

Our adherent ventral telencephalic patterning protocol was based on previous studies (Chambers et al., 2009; Li et al., 2009; Seibler et al., 2011; Ma et al., 2012; Liu et al., 2013a; Maroof et al., 2013; Nicoleau et al., 2013), and began with the 2D cultivation of a dense uniform hiPSC monolayer (Figure 2A). Neural induction was initiated by dual SMAD inhibition *via* the administration of SB-431542 and LDN-193189, inhibiting the transforming growth factor-beta (TGF-β), activin/nodal, and bone morphogenetic protein (BMP) signaling (Figure 2D; Chambers et al., 2009; Qi et al., 2017). Also, morphogens were added to the medium to achieve a rostro-ventral patterning [for rostralization the small molecule XAV-939 (XAV); for ventralization either purmorphamine (Pu) or the recombinant protein SHH] (Figure 2D). During this stage, the morphology of the cellular monolayer acquired in some parts an inner structure resembling neural rosettes (Figure 2B), which are considered to be the *in vitro* analog of the neural tube in neurogenesis (Fedorova et al., 2019). At the last stage of the protocol, the cells matured and neuronal cells with long neurites emerged (Figure 2C).

Our main focus of this study was to optimize the ventralization efficiency during rostro-ventral pattering. For this purpose, the patterning potency of the ventralizing factors purmorphamine and SHH was compared at different concentrations. Subsequently, the impact of various purmorphamine administration intervals was determined by gene expression analysis. Neuronal phenotypes were characterized by immunofluorescence stainings and the functionality was assessed by neurophysiological recordings.

### Comparison of the Rostro-Ventralization Potency Between Purmorphamine and Sonic Hedgehog

**FIGURE 3:**
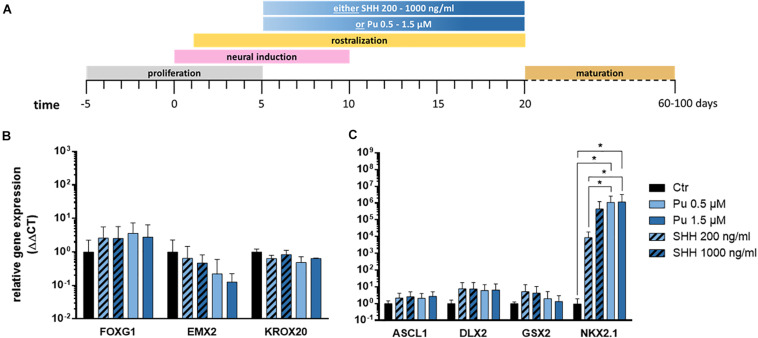
Verification of the most effective ventralization morphogen. **(A)** Differentiation protocol. Gene expression analysis of either purmorphamine- or Sonic hedgehog-treated cells relative to non-treated samples along the rostro-caudal **(B)** and dorso-ventral **(C)** axis, at day 20; *n* = three to five differentiations, three hiPSC lines. Statistical analysis: two-way ANOVA, correction for multiple testing by the Tukey method, error bars depict the SEM. Significant with *p*-value < 0.05 (*).

First, we evaluated the suitability of the small molecule purmorphamine for ventral telencephalic differentiation and thus for substitution of the recombinant protein SHH. The latter is a crucial signal peptide for the pattering and specification of the MGE *in vivo* (Xu et al., 2005). To this end, either purmorphamine (Pu) or SHH were administered for 15 days from day 5 on (Figure 3A). The concentrations were selected to promote either an LGE (Pu 0.5 μM or SHH 200 ng/ml) or an MGE (Pu 1.5 μM or SHH 1000 ng/ml) identity (Ma et al., 2012; Liu et al., 2013b). On day 20, the ventralizing efficiency was assessed using gene expression analysis. Along the rostro-caudal patterning axis (Figure 3B), the expression levels of the forebrain marker FOXG1 increased non-significantly after the exposure of both ventralization factors (Pu and SHH) compared to non-patterned cells (control; Ctr). For EMX2, a cortical marker, the expression decreased non-significantly in treated cells in a concentration-dependent manner, with a stronger effect after Pu administration than after SHH. For the rhombencephalic transcription factor KROX20, the gene expression decreased non-significantly after ventralization (Pu and SHH) compared to Ctr. Along the dorso-ventral axis (Figure 3C), the expression levels of the pan-ganglionic eminence (pan-GE) markers ASCL1 and DLX2 increased non-significantly after ventralization. Also the expression level of the LGE marker GSX2 increased after treatment, although the effect gradually diminished with higher concentrations. Strikingly, the mRNA level of the MGE marker NKX2.1 was significantly elevated in Pu-treated cells compared to Ctr and to SHH-treated (200 ng/ml) cells. Of note, the NKX2.1 expression was only slightly elevated after 1.5 μM Pu administration compared to 0.5 μM treated cells, suggesting a saturation plateau. The results of the statistical analysis are shown in Supplementary Table 5.1. Because we aimed to generate cell populations with the highest MGE identity, we focused on Pu as a ventralization factor and 0.5 μM as working concentration for the following experiments to avoid possible oversaturation effects at higher concentrations.

### Determination of Optimal Timing of Purmorphamine Administration

**FIGURE 4:**
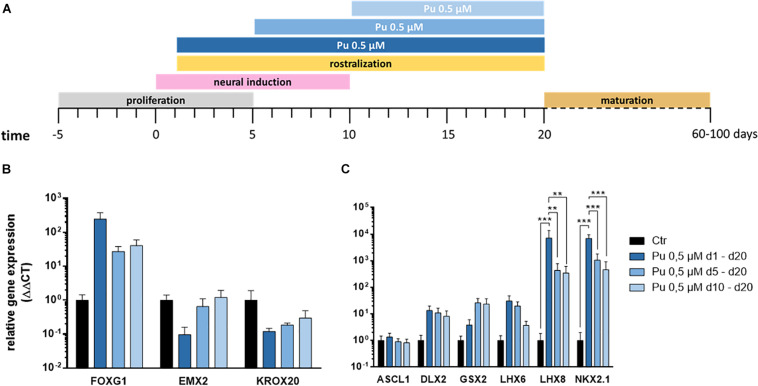
Screening for most effective timing for the application of purmorphamine. **(A)** Different time intervals for ventral patterning. After regionalization (day 20) the rostro-caudal **(B)** and dorso-ventral **(C)** identity was assessed, Pu-treated values are relative to Ctr; *n* = four differentiations, four hiPSC lines. Statistical analysis: two-way ANOVA, correction for multiple testing by the Tukey method, error bars correspond to the SEM. Significant with *p*-value < 0.005 (**), <0.0005 (***).

After verifying the suitability of Pu as a potent ventralization morphogen and its most promising concentration (0.5 μM), we aimed to determine the optimal timing for ventralization. For this purpose, three different ventral patterning time points (day 1, 5, and 10) with decreasing duration (19, 15, and 9 days) were evaluated (Figure 4A).

The corresponding gene expression analysis (day 20) revealed – along the rostro-caudal axis – for FOXG1 a non-significant increase after the earliest treatment compared to Ctr while the effect declined with later ventral regionalization (Figure 4B). In line with this, a clear time dose-effect was evident for the cortical (EMX2) and the hindbrain (KROX20) markers, as the expression decreased with earlier ventral regionalization (Figure 4B). Along the dorso-ventral axis, the cholinergic (LHX8), GABAergic (LHX6), pan-GE (DLX2), and MGE (NKX2.1) markers displayed a temporal dosage effect: longer and earlier ventralization intervals revealed higher expression levels (Figure 4C). In contrast, the LGE marker GSX2 displayed an inverse temporal dosage effect, with non-significantly higher mRNA levels after later and shorter ventralization. Strikingly, the expression levels were significantly upregulated for NKX2.1 and LHX8 after the earliest onset (d1) of rostro-ventralization compared to later treatments and Ctr. The underlying statistical analysis is listed in Supplementary Table 5.2. Notably, the above-mentioned findings could be observed in all cell lines (see Supplementary Figures 1, 2).

**FIGURE 5:**
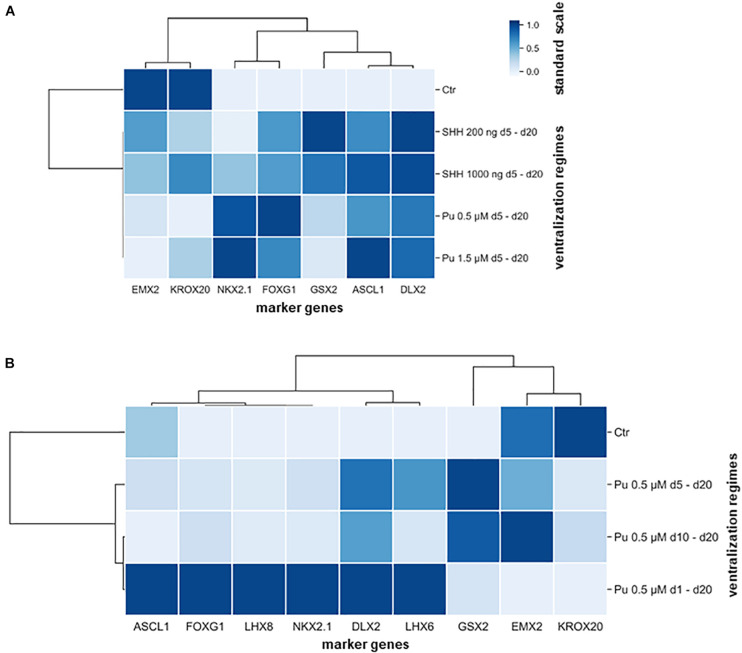
Hierarchical cluster heatmaps segregating the gene expression profiles (transverse axis) from the expression signature (longitudinal axis) according to applied rostro-ventralization regimes. **(A)** The first cluster map is based on RT-qPCR datasets from the comparison between Pu vs. SHH. The gene expression profiles were clustered into four groups: most downregulated after Pu treatment (EMX2 and KROX20), most upregulated after Pu treatment (NKX2.1 and FOXG1), most upregulated after SHH 200 ng/ml administration (GSX2), and in general upregulated after rostro-ventralization (ASCL1 and DLX2). Furthermore, the ventralization regimens appeared to cluster by morphogen type rather than by applied concentration. **(B)** The second cluster map is based on RT-qPCR datasets from different Pu treatment intervals. Here, the gene expression profiles clustered in three major groups: most elevated after the earliest and longest purmorphamine administration (d1–d20) (forebrain, pan-GE, GABAergic, and MGE-specific markers), most upregulated after the intermediate purmorphamine (d5–d20) interval (LGE); most downregulated after the earliest and longest purmorphamine administration (d1–d20) (cortical and hindbrain). Likewise, the ventralization regimes clustered whether they promoted an MGE-like expression signature or resulted in an LGE-like identity. The standard scale was calculated for each gene (column).

Subsequently, hierarchical cluster analysis was performed for each experimental setup to determine the most appropriate patterning regime for generating MGE-specific fates (Figure 5). In the first differentiation regime (Pu vs. SHH), the gene clustering (top side) diverged first EMX2 and KROX20 from all other genes (Figure 5A). When looking at the standard scale, both genes are downregulated the most after both Pu-treatments (Pu 0.5/1.5 μM). The next clade subdivided NKX2.1 and FOXG1 from the remaining ganglionic marker genes (GSX2, ASCL1, and DLX2) corresponding to their transcription level per treatment type. On the other hand, the ventralization regimes (left panel) separated into treated and untreated conditions, while the ventralizing subgroups diverged between SHH and Pu and interestingly not into higher and lower morphogen concentrations. In the second differentiation regime (Pu time intervals), the first clade first discriminated in genes that were either downregulated by the ventralization regimes (EMX2 and KROX20) or displayed a different time window for elevated expression levels (GSX2) (Figure 5B). In the other clade, the remaining genes were grouped that were most strongly expressed after the earliest and longest Pu treatment (d1–d20). Again, the ventralization regimes first clustered between treated and untreated conditions. The following clade separated the intermediate Pu treatment duration (d5–d20) from all other Pu regimes corresponding to the GSX2 expression level. The clustering revealed the importance of the correct morphogen type, as well as the patterning timing for the aimed cell identity. In general, purmorphamine appears to be more suitable to promote an MGE-like identity than SHH. In particular, the earliest and longest Pu treatment led to the most substantial enrichment of forebrain (FOXG1), pan-GE (ASCL1 and DLX2), MGE (NKX2.1), cholinergic (LHX8), and GABAergic (LHX6) markers, while cortical (EMX2) and hindbrain (KROX20) regionalization was restricted *in vitro*. Of note, in a short ventralization time window (Pu 0.5 μM or SHH 200 ng/ml d5–d20), a specific lateral ganglionic (GSX2) identity seems to be more pronounced. Upon later ventralization (Pu d10–d20), or SHH administration, cortical (EMX2) and rhombencephalic (KROX20) identities become more prominent compared to Pu d1–d20. The clustered data are listed in Supplementary Table 5.3.

In summary, the ventral patterning regime (Pu 0.5 μM d1–d20) with the earliest onset – during neural induction – and the highest Pu concentration led to the strongest upregulation of MGE, cholinergic, and GABAergic marker genes on mRNA-level.

### Qualitative Immunofluorescence Characterization After Terminal Differentiation Confirms Retention of Induced Cellular Regional Identity

**FIGURE 6:**
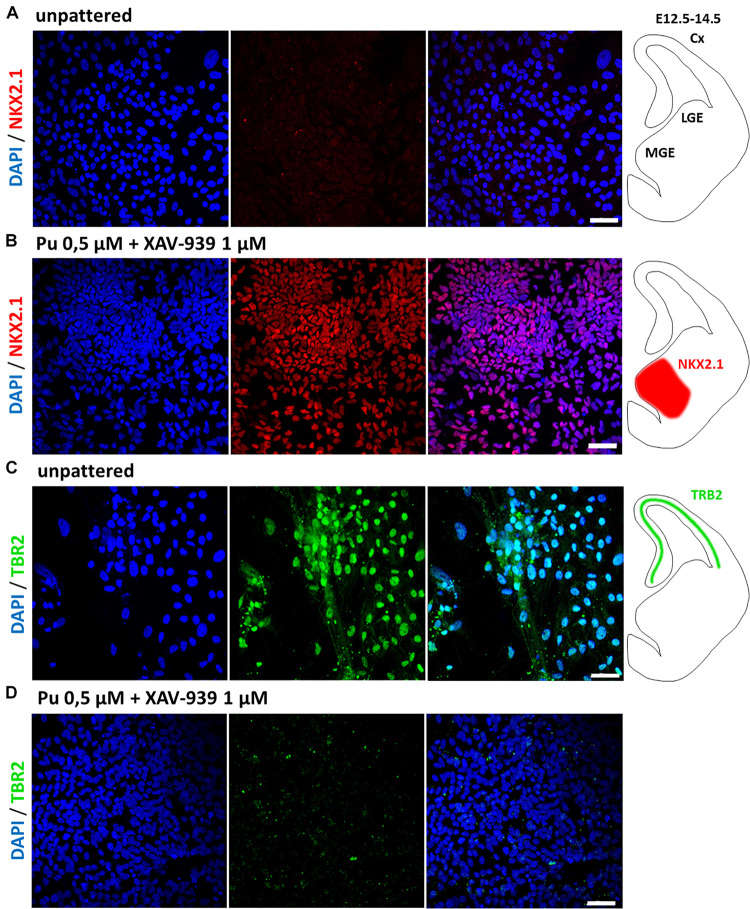
Immunofluorescence images of 55 days *in vitro* (35 days after final maturation) neuronal populations. **(A)** In non-regionalized cells, the MGE-specific marker NKX2.1 was not detected, whereas **(B)** rostro-ventralized populations retained their adopted MGE identity after final maturation. **(C)** In contrast, the unpatterned cells exhibited a cortical identity (TBR2) which could not be identified in the rostro-ventralized populations **(D)**. The first panel shows nuclear staining with DAPI (blue); the second panel shows protein detection of NKX2.1 (red) or TBR2 (green); the third panel shows the overlay of the first two panels; the last column schematically shows the expression region of NKX2.1 and TBR2 in coronal sections of the left hemisphere in a 12.5–14.5-day-old mouse embryo (Englund et al., 2005; Du et al., 2008). Cx, cortex; LGE, lateral ganglionic eminence; MGE, medial ganglionic eminence. Scale bars are 40 μm.

Having quantitatively determined the effective rostro-ventral patterning condition at the progenitor cell stage, we aimed to verify which neuronal identity the cells possessed after at least additional 30 days of final differentiation. Representative immunofluorescence images display the effect of rostro-ventralized cells in comparison to non-patterned samples. Control cells, which were only neurally induced, did not show any noticeable fluorescence staining of the MGE marker NKX2.1 (Figure 6A). In contrast, this transcription factor was extensively detected in Pu-treated cells (Figure 6B), which is in line with our expression data. The region in which NKX2.1 is present in the brain of 12.5-day-old mouse embryos (Du et al., 2008) is schematically shown in the right panel of Figure 6. Furthermore, rostro-ventral patterning resulted in a substantial reduction of TBR2 compared to unpatterned cells (Figures 6C,D). TBR2 is a transcription factor that is highly expressed in neural precursor cells of the neocortex (Englund et al., 2005). Therefore, we could verify on protein level that the patterning changed – as expected – the regional identity from a cortical to an MGE-like phenotype.

**FIGURE 7:**
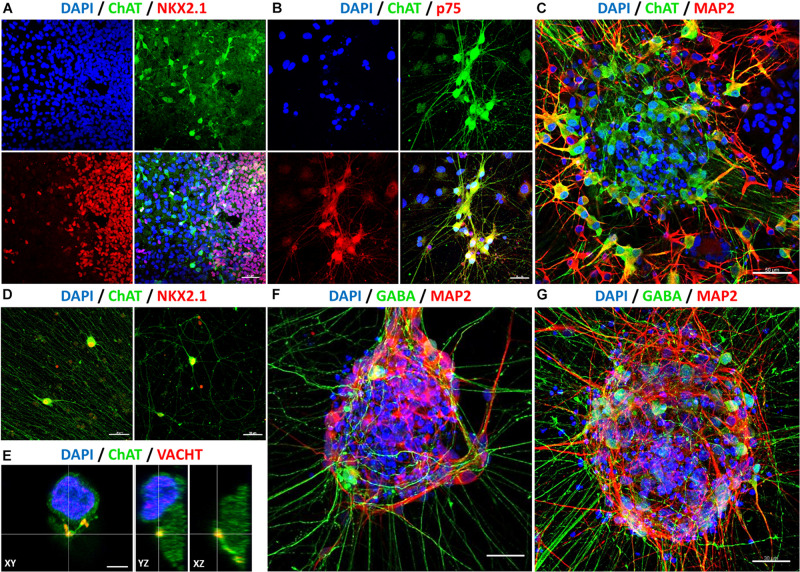
Determination of neuronal identity with immunofluorescence stainings after 60 days *in vitro* (40 days after final maturation). After rostro-ventralization, the presence of BFCNs was confirmed by coexpression of ChAT (green: **A–E**), with NKX2.1 (red: **A,D**), p75 (red: **B**), MAP2 (red: **C**), and VACHT (red: **E**). Compared to unpatterned samples **(F)**, qualitatively more GABA-positive neurons and neurites (GABA: green, MAP2: red) were observed in patterned cell populations **(G)**. Nuclei were stained with DAPI (blue). Scale bars: 40 μm **(A)**, 30 μm **(B,D,F,G)**, 50 μm **(C)**, and 5 μm **(E)**.

Further immunofluorescence stainings were performed to characterize the differentiated cells in more detail. Figure 7 shows immunostained cells harboring a BFCN identity. A proportion of NKX2.1-positive cells was also ChAT-positive (Figures 7A,D). Choline acetyltransferase (ChAT) is responsible for the biosynthesis of the neurotransmitter acetylcholine, from acetyl-coenzyme A and choline (Cho et al., 2014), and thus necessary for the formation of cholinergic interneurons. Additional evidence for a BFCN-identity is the co-expression of p75 with ChAT (Figure 7B). The neutrophin receptor p75 encodes for a surface marker of mature BFCNs (Schnitzler et al., 2008). Furthermore, the neuronal identity of ChAT-positive cells was validated by coexpression of MAP2 (Figure 7C). The microtubule-associated protein 2 is involved in microtubule stabilization (Qiang et al., 2006) and intracellular trafficking (Gumy et al., 2017); thus essential for the cytoarchitecture of neurons. The cholinergic identity was further verified by the detection of the vesicular acetylcholine transporter (VACHT) in synaptic boutons (Figure 7E).

In addition to cholinergic interneurons, GABAergic interneurons also originate from the MGE (Marın et al., 2000). In keeping with our previous expression data, we observed the appearance of GABAergic cells. Compared to non-patterned cells (Figure 7F), a much higher proportion of GABA- and MAP2-positive cells with a higher amount of projections was observed in rostro-ventralized samples (Figure 7G). Of note, cells at the periphery of cell clusters exhibited more often a mature phenotype as well as a more mature morphology (e.g., longer neurites) than cells within cell clusters (Figures 7B–D,F,G). Overall, these data lead to the notion that our hiPSC-derived MGE-like populations included cholinergic as well as GABAergic neurons with a mature basal forebrain identity.

### Electrophysiological Characterization of hiPSC-Derived Cholinergic Neurons

**FIGURE 8:**
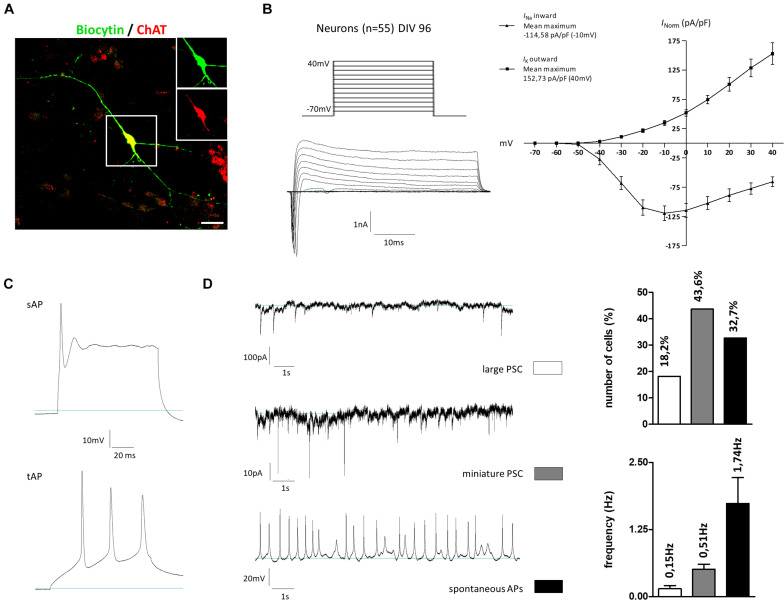
Electrophysiological properties of hiPSC-derived cholinergic neurons 96 days after the onset of differentiation. **(A)** Representative image of a large multipolar neuron (*n* = 55) that was selected for patch-clamp studies. For later immunotype characterization some of the recorded cells were marked *via* biocytin and subsequent streptavidin staining. Insets show biocytin (green) and ChAT (red) staining in the perinuclear region. Scale bar: 20 μm. **(B)** Depolarizing steps in stepwise increments of 10 mV from a holding potential of –70 to +40 mV were performed in the whole-cell voltage-clamp mode to trigger voltage-dependent sodium and potassium currents. **(C)** Evoked action potentials (AP) were recorded as single APs (sAP) but also as train APs (tAP) typically observed in mature neurons. **(D)** Spontaneous activity was observed in the whole-cell recording mode by means of large AP-dependent postsynaptic currents (large PSC; upper figure) in 18.2% of the cells with a frequency of 0.15 ± 0.06 Hz. As a result of spontaneous vesicle release, miniature PSCs were recorded in 43.6% of all recorded cells with a frequency of 0.51 ± 0.09 Hz. Current-clamp recordings further revealed the firing of spontaneous APs in 32.7% of the cells with a frequency of 1.74 ± 0.48 Hz. All values are presented as means ± SEM.

To investigate the neuronal activity of our generated cells, whole-cell patch-clamp measurements were performed on cells kept for 96 days *in vitro*. The selection criterion for recordings was the morphology of the cells, which should correspond to that of cholinergic neurons: a fusiform soma, with a diameter of 20–50 μm, giving rise to thick dendrites, which branched into finer processes of higher orders. The cholinergic identity of some recorded cells was confirmed by *post hoc* immunostainings: neurons on two coverslips were filled during recording this way (∼5 neurons per coverslip). After fixation and incubation, three of these neurons could still be identified. All were positive for ChAT as well (Figure 8A). In the first measurement, a current-voltage relation was determined (Figure 8B). For this, holding potentials were increased in increments of 10 mV steps from −70 to +40 mV. The presence of voltage-gated potassium outward and sodium inward channels was shown by short influxes (sodium) and long effluxes (potassium) of cations after depolarization. The current maxima were normalized according to the cell membrane capacitance (pA/pF). Concerning the potassium outward current, the highest current density average (152.73 pA/pF) was measured at a membrane potential of 40 mV. The highest current density average for sodium influx was −114.58 pA/pF at an applied voltage of –10 mV. In addition to the current-voltage plot, we characterized the action potentials and synaptic activity of the cells. Besides single action potentials (sAP), trains of APs (tAP) were also monitored, which are a characteristic of mature neurons (Figure 8C). Large postsynaptic currents (PSCs) were observed in 18.2% of all measured cells with a frequency of 0.15 ± 0.06 Hz (Figure 8D). These PSCs were AP-dependent, indicating signal transductions from inter-cell communication. Miniature PSCs triggered by spontaneous vesicle releases were recorded in 43.6% of all measured cells (Figure 8D) with a frequency of 0.51 ± 0.09 Hz. Finally, spontaneous APs were also observed in 32.7% of the cells, which had a frequency of 1.74 ± 0.48 Hz (Figure 8D). Active and passive membrane properties are summarized in Supplementary Table 5.4. In sum, the gene expression pattern and immunochemical stainings contribute to the notion that our newly established protocol enables the generation of BFCN- and GABAergic-like neurons from hiPSC. In addition, the functional maturity of the neuronal population was confirmed by electrophysiological measurements.

## Discussion

Human *in vitro* models, such as hiPSC-derived BFCNs, provide an excellent platform to investigate neurological disorders, such as Alzheimer’s disease or isolated dystonias. However, the number, as well as the physiological maturity of BFCNs, differentiated from human sources is limited and the regional identity of the generated neurons is not well characterized (Ochalek et al., 2016). Therefore, we systematically tested for the most effective striatal regionalization parameters (mimicking MGE environment during embryogenesis) to evaluate MGE-specific gene expression.

Before the regionalization was initiated, neural cell fate was induced using dual SMAD inhibition (Chambers et al., 2009). When NPCs are not further regionalized, they acquire a telencephalic identity, regardless of the protocol used to initiate neural induction (Kim et al., 2014). This observation is consistent with the assumption that the first NPCs have a dorsal-telencephalic identity *a priori* during neural induction in vertebrates (Muñoz-Sanjuán and Brivanlou, 2002). These non-regionalized cells served as reference cells for gene expression and immunofluorescence analyses. As shown in other publications, telencephalic identity can be further enhanced by rostralization when the Wnt signaling pathway is blocked *via* the small molecule XAV-939 (Maroof et al., 2013) or *via* the recombinant protein DKK1 (Eiraku et al., 2008; Nicholas et al., 2013). Also, these neural progenitors can be further regionalized ventrally or dorsally. The ventral specification requires stimulation of the SHH pathway in addition to Wnt pathway inhibition. This can be facilitated either *via* the administration of recombinant SHH, or small molecules like purmorphamine (Watanabe et al., 2005; Li et al., 2009; Liu et al., 2013b; Maroof et al., 2013; Nicholas et al., 2013). In contrast to the protocols mentioned above, we aimed to establish an efficient rostro-ventralization patterning protocol suitable for hiPSC; omitting the usage of murine- or human-derived embryonal stem cells.

During the first patterning regime, cells were ventralized with either Pu (0.5–1.5 μM) or SHH (200–1000 ng/ml) 5 days after the initiation of neural induction and rostralization. Two weeks later, we assessed the regionalization along the rostro-caudal and dorso-ventral neuraxes by comparing the gene expression of different marker genes. Evaluation of the expression profiles showed that both morphogens increased the forebrain and ganglionic eminence identity, although only samples after Pu administration showed a significant increase in the MGE-specific marker NKX2.1. Since the higher Pu concentration compared to the lower concentration did not result in a significant upregulation of NKX2.1, we focused on Pu 0.5 μM for the determination of the most favorable timing during ventralization.

To assess when the ventralization patterning is most effective, purmorphamine was administered either at onset, during, or after neural induction. The expression levels of MGE-markers correlated with earlier and longer regionalization time intervals. This finding corresponds to the assumption, that dosage and temporal coordination have a crucial role in ventral identity expression (Fasano et al., 2010). In contrast, expression levels of EMX2 (cortical), KROX20 (hindbrain), as well as GSX2 (LGE), increased with later regionalization. Strikingly, we could confirm the abovementioned observations by employing unsupervised hierarchical linkage clustering of our gene expression data sets. Thus, we confirmed that different optimal time intervals exist for specific regionalizations with the MGE possessing an earlier time window than LGE. In conclusion, the substantial increase in telencephalic ventral markers NKX2.1, LHX6, and LHX8 after the conducted patterning reflects the regionalization of our human-derived cells into a MGE identity, potentially reflecting the specified *in vivo* neurogenesis *in vitro*.

When comparing the relative gene expression levels between the two experimental setups, there are – in some cases – noticeable differences for the same genes. Since the controls were not patterned, spontaneous differentiation occurred, the direction of which could not be influenced. In particular, for controls, the expression levels of LHX8 and NKX2.1 were sometimes detectable only at very late CT values (see Supplementary Figure 1). On the other hand, the expression levels within each treatment condition of the target genes varied mostly within one to two logarithmical scales across all cell lines (see Supplementary Figure 2), demonstrating that the collected gene expression data are robust within the experimental setups. With this, we explain the divergent relative gene expression levels for some genes between both setups.

After the most efficient rostro-ventral patterning protocol for the progenitor cell stage was evaluated, the identity of matured cells was qualitatively determined in comparison to non-regionalized cells by immunofluorescence analysis. Remarkably, the MGE-like identity remained after regionalization – with nearly homogenous NKX2.1 expression, whereas non-patterned cells were negative for NKX2.1. In contrast, the control cells exhibited a cortical phenotype (high number of TBR2-positive cells), whereas this phenotype was virtually absent in patterned cells. This indicates that the cells retained their cell fate, as previously determined by gene expression analysis. Upon detailed characterization of the MGE identities, we found that a large number of NKX2.1 positive cells co-expressed ChAT, p75, and MAP2 (cholinergic neurons) or GABA and MAP2 (presumably GABAergic interneurons). Therefore, we verified the phenotype of BFCNs and GABAergic neurons (Liu et al., 2013b), which was previously validated on the mRNA level. By simultaneously initiating a neural and rostro-ventral cell fate, we were able to facilitate an MGE-like niche *in vitro* that served as a reservoir for the development of BFCNs and GABAergic interneurons, thereby specifically mimicking the *in vivo* neurogenesis of these cell types (Sussel et al., 1999). However, we found that the majority of cells showing a mature phenotype to be positioned at the periphery of cell clusters, whereas in cell clusters mature identities were detected to a lesser extent. A possible reason for this observation could be that the cell clusters serve as proliferative niches, from where the cells emigrate and eventually mature. Ultimately, this is consistent with the *in vivo* differentiation of striatal interneurons, which develop from the MGE. As differentiation progresses in the embryonic brain, the neurons migrate and mature into the striatum and cortex, respectively (Marın et al., 2000).

Finally, we showed that our differentiated MGE-like cells generated action potentials and synaptic activity. The sodium and potassium current amplitudes corresponded to forebrain interneurons differentiated from 8 to 12 weeks (Nicholas et al., 2013). Furthermore, we observed that 18% of cells had large PSCs and 44% of cells showed miniature PSCs. The generation of spontaneous action potentials was observed in 33% of the cells. Collectively, these functional data are indications for electrophysiologically active cells. However, the percentage of PSC- and spontaneous tAP-positive cells may increase after prolonged maturation (Nicholas et al., 2013). Besides, the co-cultivation of glia cells may also lead to higher synapse density and elevated electrophysical activity (Boehler et al., 2007; Nicholas et al., 2013). Further studies could establish CRISPR-Cas9-generated BFCN-reporter lines, which would enable a precise electrophysiological analysis of the corresponding cell type.

In contrast to recent studies, our patterning regime omitted the use of non-adherent approaches, while simultaneously inhibiting the Wnt-pathway during neuralization and ventralization (Ihnatovych et al., 2018; Muñoz et al., 2020). Plating cells under adherent conditions with constant numbers, increase the reproducibility and uniformity of the differentiation experiments in contrast to the formation of embryoid bodies, a process that is more difficult to control. Although more straightforward to operate to non-adherent protocols, we were able to obtain high enrichments of MGE-like neuronal populations. These neuronal populations showed electrophysiological activities (e.g., spontaneous APs) of mature neurons whose cholinergic identity could be verified by *post hoc* stainings. Therefore, our approach might be more favorable for future high-throughput examinations compared to non-adherent protocols.

In summary, the timing and the stimulation strength of the SHH pathway are crucial for the emergence of ventral telencephalic identities, like BFCNs and GABAergic interneurons. Therefore, rostro-ventral regionalization should be performed in parallel with neural induction.

## Materials and Methods

### Expansion of Induced Pluripotent Stem Cell

As previously described (Seibler et al., 2011; Pauly et al., 2018), hiPSCs were generated from primary dermal fibroblasts. Briefly, fibroblasts were transduced with retroviral vectors to overexpress OCT4, SOX2, cMYC, and KLF4. In total, four hiPSC lines from healthy adult donors (who had given informed consent according to the ethical regulations of the University of Lübeck) were employed for the investigations (see Supplementary Table 3). HiPSC colonies were cultured on irradiated mouse embryonic fibroblasts (MEFs) in hiPSC medium (see Supplementary Table 1.1) and harvested by dissociation with Accutase (Gibco, Carlsbad, CA, United States) for 15 min. For adherent neural induction (Chambers et al., 2009), 120,000 cells/cm^2^ were transferred to Matrigel-coated 24-well culture plates (Corning, Corning, NY, United States) (Figure 2D). To facilitate the switch from MEF feeder layer to feeder layer-free/Matrigel-based cultivation, hiPSCs were expanded with MEF-conditioned hiPSC medium (CM). In addition, the CM was supplemented with FGF-2 (5 ng/ml; Merck Millipore, Darmstadt, Germany) and the ROCK-inhibitor Y-27632 (10 μM; Stemcell Technologies, Vancouver, BC, Canada) for increased cell survival (Watanabe et al., 2007). The generation of CM was described here (Tomishima, 2008). In short, MEFs were plated onto 10 cm^2^ dishes (at 50,000 cells/cm^2^ density) with DMEM supplemented with 10% fetal bovine serum (HyClone, Cytiva, Marlborough, MA, United States). The next day, the medium was removed and cells were washed with 1× PBS (Gibco, Carlsbad, CA, United States). Subsequently, 10 ml hiPSC medium per dish were applied. After 24 h, the CM was collected and stored at −20°C until use. The conditioning was carried out twice per MEF plate (in total 20 ml CM per plate).

### Neural Induction, Rostro-Ventralization, and Maturation

The following section is illustrated in Figure 2D. When the expanded hiPSCs reached 80% confluency, neural induction (day 0) was initiated by the addition of the small molecules SB-431542 (10 μM; Tocris, Bristol, United Kingdom) as a TGF-β inhibitor and LDN-193189 (100 nM; Stemgent, Cambridge, MA, United States) as a BMP-inhibitor in KSR medium. From day 5 to 10, the KSR medium was progressively replaced every other day with the N2B27 medium (KSR:N2B27 volume:volume ratio in percent; day 5–6 75:25, day 7–8 50:50, day 8–9 25:75, and day 10 0:100) while Y-27632 and SB-431542 were withdrawn. The composition of KSR and N2B27 are listed in Supplementary Tables 1.2, 1.3. For the rostro-ventral patterning experiments, cells were treated with XAV-939 (day 1–20) (1 μM; Stemgent, Cambridge, MA, United States) and with either SHH (day 5–20) (200 or 1000 ng/ml; R&D Systems, Minneapolis, MN, United States) or with varying concentrations (0.5 or 1.5 μM) and durations (day 1/5/10–20) of purmorphamine (Stemgent, Cambridge, MA, United States). The patterning steps were omitted in controls. On day 10, the cells (preferably neural rosettes) were transferred *en bloc* (approximately 1 mm edge length) to Matrigel-coated 12-well culture plates, and Y-27632 (10 μM) was added for 24 h. Again, on day 20, samples were transferred *en bloc* onto poly-D-lysine- (Sigma-Aldrich, St. Louis, MO, United States) and laminin-coated (Roche, Basel, Switzerland) coverslips (Hecht, Sondheim, Germany) into 12-well culture plates (Corning, Corning, NY, United States). A subset of cells was employed for gene expression analysis. The plated cells were terminally matured for at least 30 days until immunofluorescence stainings and electrophysiological measurements in N2B27 medium containing BDNF (20 ng/ml; PeproTech, Hamburg, Germany), GDNF (10 ng/ml; PeproTech, Hamburg, Germany), IGF1 (10 ng/ml; PeproTech, Hamburg, Germany), db-cAMP (500 μM; Enzo Life Sciences, Farmingdale, NY, United States), and DAPT (10 μM; Tocris, Ellisville, MO, United States). The first three factors are neurotrophins that increased survival and supported neural maturation (Liu et al., 2013a). The last two factors supported the formation of a mature neural morphology (Kirkeby et al., 2012). Of note, the quality of the generated cells was determined morphologically at least every other day. The proportion of neural rosette-like structures was monitored during rostro-ventral specification. Until the second passage at the latest, an experimental run was rejected if much of the population morphology did not correspond to a dense monolayer with an internal structure resembling neural rosettes (excluding controls).

### Gene Expression Analysis

The RNA extraction and RT-qPCR were conducted as described recently (Pauly et al., 2018; Stengel et al., 2020). In short, cell samples were homogenized (QIAshredder kit, Qiagen, Hilden, Germany) and followed by purification of total RNA (RNeasy Mini Kit, Qiagen, Hilden, Germany). Subsequently, RNA was transcribed to cDNA by the Maxima First Strand cDNA Synthesis Kit for RT-qPCR (Thermo Fisher Scientific, Waltham, MA, United States). Relative gene expression levels were analyzed using the comparative ΔΔCT method by regular RT-qPCR following the manufacture’s protocol for LightCycler FastStart DNA Master SYBR Green I Kit and LightCycler^480^ (Roche, Basel, Switzerland) (for the primer sequence see Supplementary Table 2). Unsupervised hierarchical gene expression and ventralization regime clustering were performed using seaborn (0.11.1) (Waskom, 2021) and the complete linkage method, while the standard scale was calculated for the genes (columns).

### Immunofluorescence Staining

Cells were treated as recently described (Stengel et al., 2020). In detail, cells were washed with 1× PBS (Gibco, Carlsbad, CA, United States) and then fixed with 4% paraformaldehyde (Sigma-Aldrich, St. Louis, MO, United States) for 30 min at room temperature. After repeated washing steps with PBS, non-specific binding sites were blocked for 45 min with PBS containing 5% normal donkey (Sigma-Aldrich, St. Louis, MO, United States) or goat serum (Invitrogen, Carlsbad, CA, United States), 0.1% Triton X-100 (AppliChem, Darmstadt, Germany) and 0.01% NaN_3_ (Sigma-Aldrich, St. Louis, MO, United States). Primary antibodies were incubated overnight at 4°C, followed by a washing step (1 × 15 min) with washing buffer (PBS with 0.1% Triton X-100). Secondary antibodies were incubated for 2 h at room temperature (the employed antibodies are listed in Supplementary Table 4). Both primary and secondary antibodies were diluted in PBS with 1% normal donkey or goat serum containing 0.1% Triton X-100 and 0.01% NaN_3_. Subsequently, the samples were quenched using Sudan Black (0.1%, in 70% ethanol; Sigma-Aldrich, St. Louis, MO, United States) for 15 min at room temperature. This step reduced autofluorescence by lipofuscin, lipids, triglycerides, and lipoproteins (Oliveira et al., 2010). Subsequently, cells were washed (3 × 5 min with wash buffer and 1 × 5 min with PBS). Finally, the samples were mounted with Vectashield containing DAPI (Thermo Fisher Scientific, Waltham, MA, United States) on slides (Menzel Gläser, Braunschweig, Germany). Samples were stored in the dark at 4°C until analysis. Imaging was performed using a confocal laser scanning microscope (LSM 710, ZEN black software; Zeiss, Jena, Germany).

### Electrophysiology

Electrophysiological measurements were employed as described previously (Stanslowsky et al., 2014). In brief, patch pipettes, made of borosilicate glass (Science Products, Hofheim, Germany), were pulled using a P-1000 (Sutter Instruments, Novato, CA, United States) and filled with an internal solution consisting of 153 mM KCl, 1 mM MgCl_2_, 10 mM HEPES, 5 mM EGTA, and 2 mM Mg-ATP, adjusted to pH 7.3 with KOH (305 mOsm) resulting in a resistance of 3–4 MΩ. The bath solution contained 142 mM NaCl, 8 mM KCl, 1 mM CaCl_2_, 6 mM MgCl_2_, 10 mM glucose, and 10 mM HEPES, adjusted to pH 7.4 with NaOH (325 mOsm). Cell fillings were performed comparable to a previous study (Capetian et al., 2018). During cell recordings on two coverslips, biocytin was included in the pipette solution to identify the phenotype of the recorded neurons by fluorescence microscopy. After recordings, the cells were subsequently fixed and streptavidin and immunofluorescence staining against the cholinergic marker ChAT was done. Whole-cell patch-clamp measurement was performed at room temperature using an inverted microscope (Zeiss, Jena, Germany). Only cells with a leakage current of <100 pA were used for the study. The recordings were filtered by a low-pass filter at 2.9 kHz, digitized *via* an EPC-10 amplifier (HEKA, Lambrecht, Germany) at 10 kHz, and analyzed using Patch Master software (HEKA).

### Statistical Analysis

Data were analyzed using GraphPad Prism 6 (GraphPad Software, San Diego, CL, United States). The patterning effect at the mRNA level was compared with a two-factorial analysis of variance (two-way ANOVA), followed by Tukey test for multiple comparisons. The means of expression levels were calculated, along with the standard error of the mean (SEM). Results were considered significant with *p*-values < 0.05, adjusted *p*-values, and row statistics of the gene expression analyses are shown in Supplementary Tables 5.1, 5.2.

## Data Availability Statement

The original contributions presented in the study are included in the article/Supplementary Material, further inquiries can be directed to the corresponding author/s.

## Ethics Statement

The studies involving human participants were reviewed and approved by the Universität zu Lübeck, Ethikkommission, Lübeck, Germany. The participants provided their written informed consent to participate in this study.

## Author Contributions

VK, FW, and PC: concept and design, data acquisition, analysis and interpretation, and manuscript writing. MN and NS: data acquisition, analysis, and interpretation (electrophysiology). MP and FS: data acquisition, analysis, and interpretation. BM: data acquisition. PS and CK: concept and design, and interpretation. All authors involved in revising the manuscript for important intellectual content and approved the final version to be published.

## Conflict of Interest

The authors declare that the research was conducted in the absence of any commercial or financial relationships that could be construed as a potential conflict of interest.

## Publisher’s Note

All claims expressed in this article are solely those of the authors and do not necessarily represent those of their affiliated organizations, or those of the publisher, the editors and the reviewers. Any product that may be evaluated in this article, or claim that may be made by its manufacturer, is not guaranteed or endorsed by the publisher.
