# Supplementary material for: Ventral Telencephalic Patterning Protocols for Induced Pluripotent Stem Cells
Source: Front Cell Dev Biol. 2021 Aug 18;9:716249. doi: 10.3389/fcell.2021.716249 (PMC8416478; doi:10.3389/fcell.2021.716249)
Supplement: Supplementary file 1 [file Table_1.DOCX]

Supplementary Material

# Composition of culture medium

## HiPSC medium, also used for conditioning by mouse embryonic fibroblasts (conditioned medium, CM)

| KnockOut™ DMEM/F-12 (1X) | 400 ml | Gibco, Carlsbad, US |
| --- | --- | --- |
| KnockOut™ Serum Replacement | 100 ml | Gibco, Carlsbad, US |
| HEPES buffer 1M | 7,5 ml | Gibco, Carlsbad, US |
| L-Glutamine 200 mM | 5 ml | Gibco, Carlsbad, US |
| MEM NEAA 100x | 5 ml | Gibco, Carlsbad, US |
| 2-Mercaptoethanol 50 mM | 1 ml | Gibco, Carlsbad, US |

## Knock-out serum replacement (KSR) medium

| KnockOut™ DMEM/F-12 (1X) | 425 ml | Gibco, Carlsbad, US |
| --- | --- | --- |
| KnockOut™ Serum Replacement | 75 ml | Gibco, Carlsbad, US |
| L-Glutamine 200 mM | 5 ml | Gibco, Carlsbad, US |
| MEM NEAA 100x | 5 ml | Gibco, Carlsbad, US |
| Pen/Strep | 5 ml | Gibco, Carlsbad, US |
| 2-Mercaptoethanol 50 mM | 1 ml | Gibco, Carlsbad, US |
| Fungizone | 500 µl | Invitrogen, Carlsbad, US |

## N2B27 medium

| Neurobasal® Medium (1X) | 250 ml | Life Technologies, Carslbad, US |
| --- | --- | --- |
| KnockOut™ DMEM/F-12 (1X) | 250 ml | Gibco, Carlsbad, US |
| L-Glutamine 200 µM | 5 ml | Gibco, Carlsbad, US |
| HEPES buffer 1M | 5 ml | Gibco, Carlsbad, US |
| N2-Supplement (100X) | 5 ml | Gibco, Carlsbad, US |
| NeuroCult™ SM1 Neuronal Supplement | 10 ml | Stemcell Technologies, Vancouver, CA |
| Pen/Strep | 5 ml | Gibco, Carlsbad, US |
| MEM NEAA 100x | 5 ml | Gibco, Carlsbad, US |

# List of primer pairs

| Gene | Forward Primer | Reverse Primer |
| --- | --- | --- |
| ASCL1 | CAT CTC CCC CAA CTA CTC CA | AAC GCC ACT GAC AAG AAA GC |
| DLX2 | GCA CAT GGG TTC CTA CCA GT | TTG GCT TCC CGT TCA CTA TC |
| EMX2 | ACC TTC TAC CCC TGG CTC AT | GCC CAC CAC GTA GTG ATT CT |
| FOXG1 | TGT TGA CTC AGA ACT CGC TGG | CTG CTC TGC GAA GTC ATT GAC |
| GSX | TTA AGG GCC AGT TCT CTT CG | CTC CGG AGT CGA GAC AGG TA |
| KROX0 | CCA AGG CCG TAG ACA AAA TC | GGA TAT GGG AGA TCC AAC GA |
| LHX6 | GCA GCA GAA CAG CTG CTA CA | CAG AGC ACC TTC TCC TCG AC |
| LHX8 | CAA GCA CAA TTT GCT CAG GA | GGC ACG TAG GCA GAA TAA GC |
| NKX2.1 | GAC ACC ATG AGG AAC AGC | ACA GGT ACT TCT GTT GCT TG |
| β-actin | TGA AGT GTG ACG TGG ACA TC | GGA GGA GCA ATG ATC TTG AT |

# Applied hiPSC lines

| Sample ID | Sex | Age at biopsy | Clinical  status | Biopsy  source | Reprogramming method* |
| --- | --- | --- | --- | --- | --- |
| 2130 | ♂ | 38 | unaffected | fibroblasts | retroviral |
| 2131 | ♂ | 59 | unaffected | fibroblasts | retroviral |
| 2132 | ♀ | 33 | unaffected | fibroblasts | retroviral |
| 2135 | ♂ | 40 | unaffected | fibroblasts | retroviral |

*Seibler, P., Graziotto, J., Jeong, H., Simunovic, F., Klein, C., and Krainc, D. (2011). Mitochondrial Parkin recruitment is impaired in neurons derived from mutant PINK1 induced pluripotent stem cells. *J. Neurosci.* 31, 5970–5976. doi: 10.1523/JNEUROSCI.4441-10.2011

# List of antibodies

## Primary antibodies

Antigen Isotype Dilution Catalogue number Manufacture

ChAT goat 1:1000 AB144P Sigma Aldrich, St. Louis, USA

GABA rabbit 1:1000 A2052 Sigma Aldrich, St. Louis, USA

MAP2A, 2B mouse 1:800 MAB378 Sigma Aldrich, St. Louis, USA

NKX2.1 mouse 1:800 MAB5460 Sigma Aldrich, St. Louis, USA

p75 rabbit 1:2000 G3231 Promega, Fitchburg, USA

TBR2 rabbit 1:800 ab23345 abcam, Cambridge, GB

VACHT rabbit 1:1000 ab235201 abcam, Cambridge, GB

## Secondary antibodies

Antigen Isotype Dilution Flurochrome Catalogue number Manufacture

goat IgG donkey 1:800 Alexa 594 A-11058 Life Technologies, Carlsbad, USA

rabbit IgG donkey 1:800 Alexa 568 A-10042 Life Technologies, Carlsbad, USA

rabbit IgG goat 1:1000 Alexa 488 A-11008 Life Technologies, Carlsbad, USA

mouse IgG donkey 1:800 Alexa 488 A-21202 Life Technologies, Carlsbad, USA

# Statistics

## A) Relative expression levels ΔΔCT of marker genes after purmorphamine (Pu) 0.5/1.5 µM d5 - d20 vs. Sonic hedgehog (SHH) 200/1000 ng/ml treatment. B) Statistical significance determined using two-way ANOVA, corrected for multiple testing by Tukey test, alpha=5%.

| **A** | **Row statistics** | | | | | | | | | | | | | | |
| --- | --- | --- | --- | --- | --- | --- | --- | --- | --- | --- | --- | --- | --- | --- | --- |
|  | **Ctr** | | | **Pu 0.5 µM** | | | **Pu 1.5 µM** | | | **SHH 200 ng/ml** | | | **SHH 1000 ng/ml** | | |
| **Genes** | Mean | SEM | N | Mean | SEM | N | Mean | SEM | N | Mean | SEM | N | Mean | SEM | N |
| **FOXG1** | 1.00E+00 | 7.12E-01 | 3 | 3.59E+00 | 2.16E+00 | 3 | 2.78E+00 | 2.11E+00 | 3 | 2.58E+00 | 1.72E+00 | 3 | 2.54E+00 | 1.81E+00 | 3 |
| **EMX2** | 1.00E+00 | 7.19E-01 | 3 | 2.21E-01 | 2.17E-01 | 3 | 1.26E-01 | 5.70E-02 | 3 | 6.42E-01 | 4.70E-01 | 3 | 4.67E-01 | 2.02E-01 | 3 |
| **KROX20** | 9.99E-01 | 1.26E-01 | 3 | 4.86E-01 | 1.35E-01 | 3 | 6.40E-01 | 1.04E-02 | 3 | 6.31E-01 | 9.60E-02 | 3 | 8.37E-01 | 1.68E-01 | 3 |
| **ASCL1** | 1.00E+00 | 2.55E-01 | 3 | 2.07E+00 | 1.12E+00 | 3 | 2.70E+00 | 1.34E+00 | 3 | 2.14E+00 | 1.13E+00 | 3 | 2.55E+00 | 1.39E+00 | 3 |
| **DLX2** | 9.99E-01 | 3.58E-01 | 3 | 6.04E+00 | 4.29E+00 | 3 | 6.50E+00 | 4.93E+00 | 3 | 7.59E+00 | 5.82E+00 | 3 | 7.42E+00 | 6.34E+00 | 3 |
| **GSX2** | 1.00E+00 | 1.39E-01 | 3 | 1.98E+00 | 1.88E+00 | 3 | 1.32E+00 | 9.57E-01 | 3 | 5.13E+00 | 4.74E+00 | 3 | 4.23E+00 | 3.59E+00 | 3 |
| **NKX2.1** | 9.48E-01 | 4.44E-01 | 5 | 1.09E+06 | 7.49E+05 | 4 | 1.15E+06 | 9.04E+05 | 5 | 8.70E+03 | 4.58E+03 | 5 | 4.37E+05 | 3.42E+05 | 5 |

| **B** | **Adjusted P Values** | | | | | | |
| --- | --- | --- | --- | --- | --- | --- | --- |
| **Treatments** | **FOXG1** | **EMX2** | **KROX20** | **ASCL1** | **DLX2** | **GSX2** | **NKX2.1** |
| **Ctr vs. Pu 0.5 µM** | > 0,9999 | > 0,9999 | > 0,9999 | > 0,9999 | > 0,9999 | > 0,9999 | 0,0428 |
| **Ctr vs. Pu 1.5 µM** | > 0,9999 | > 0,9999 | > 0,9999 | > 0,9999 | > 0,9999 | > 0,9999 | 0,0156 |
| **Ctr vs. SHH 200 ng/ml** | > 0,9999 | > 0,9999 | > 0,9999 | > 0,9999 | > 0,9999 | > 0,9999 | > 0,9999 |
| **Ctr vs. SHH 1000 ng/ml** | > 0,9999 | > 0,9999 | > 0,9999 | > 0,9999 | > 0,9999 | > 0,9999 | 0,7399 |
| **Pu 0.5 µM vs. Pu 1.5 µM** | > 0,9999 | > 0,9999 | > 0,9999 | > 0,9999 | > 0,9999 | > 0,9999 | 0,9997 |
| **Pu 0.5 µM vs. SHH 200 ng/ml** | > 0,9999 | > 0,9999 | > 0,9999 | > 0,9999 | > 0,9999 | > 0,9999 | 0,0454 |
| **Pu 0.5 µM vs. SHH 1000 ng/ml** | > 0,9999 | > 0,9999 | > 0,9999 | > 0,9999 | > 0,9999 | > 0,9999 | 0,4384 |
| **Pu 1.5 µM vs. SHH 200 ng/ml** | > 0,9999 | > 0,9999 | > 0,9999 | > 0,9999 | > 0,9999 | > 0,9999 | 0,0167 |
| **Pu 1.5 µM vs. SHH 1000 ng/ml** | > 0,9999 | > 0,9999 | > 0,9999 | > 0,9999 | > 0,9999 | > 0,9999 | 0,2762 |
| **SHH 200 ng/ml vs. SHH 1000 ng/ml** | > 0,9999 | > 0,9999 | > 0,9999 | > 0,9999 | > 0,9999 | > 0,9999 | 0,7539 |

## A) Relative expression levels ΔΔCT of marker genes after divergent ventralization intervals Pu (0.5 µM d1/5/10- d20). B) Statistical significance determined using two-way ANOVA, corrected for multiple testing by Tukey test. alpha=5%.

| **A** | **Row statistics** | | | | | | | | | | | |
| --- | --- | --- | --- | --- | --- | --- | --- | --- | --- | --- | --- | --- |
|  | **Ctr** | | | **Pu 0.5 µM d1 - d20** | | | **Pu 0.5 µM d5 - d20** | | | **Pu 0.5 µM d10 - d20** | | |
| **Genes** | Mean | SEM | N | Mean | SEM | N | Mean | SEM | N | Mean | SEM | N |
| **FOXG1** | 1.00E+00 | 4.50E-01 | 4 | 2.46E+02 | 1.34E+02 | 4 | 2.72E+01 | 1.09E+01 | 4 | 4.05E+01 | 1.88E+01 | 4 |
| **EMX2** | 1.00E+00 | 4.19E-01 | 4 | 9.65E-02 | 6.44E-02 | 4 | 6.51E-01 | 4.57E-01 | 4 | 1.21E+00 | 7.24E-01 | 4 |
| **KROX20** | 1.00E+00 | 9.15E-01 | 4 | 1.20E-01 | 2.76E-02 | 4 | 1.86E-01 | 2.68E-02 | 4 | 2.98E-01 | 1.96E-01 | 4 |
| **ASCL1** | 1.00E+00 | 4.80E-01 | 4 | 1.35E+00 | 5.26E-01 | 4 | 9.04E-01 | 2.53E-01 | 4 | 8.19E-01 | 3.10E-01 | 4 |
| **DLX2** | 1.00E+00 | 5.65E-01 | 4 | 1.34E+01 | 6.41E+00 | 4 | 1.08E+01 | 5.56E+00 | 4 | 8.18E+00 | 4.58E+00 | 4 |
| **GSX2** | 1.00E+00 | 4.49E-01 | 4 | 3.82E+00 | 2.14E+00 | 4 | 2.58E+01 | 1.19E+01 | 4 | 2.34E+01 | 1.37E+01 | 4 |
| **LHX6** | 9.99E-01 | 5.08E-01 | 4 | 3.05E+01 | 1.71E+01 | 4 | 1.96E+01 | 8.90E+00 | 4 | 3.72E+00 | 1.56E+00 | 4 |
| **LHX8** | 9.99E-01 | 8.36E-01 | 4 | 7.06E+03 | 6.51E+03 | 4 | 4.33E+02 | 3.38E+02 | 4 | 3.45E+02 | 2.65E+02 | 4 |
| **NKX2.1** | 1.00E+00 | 9.98E-01 | 4 | 6.87E+03 | 2.54E+03 | 4 | 1.05E+03 | 7.48E+02 | 4 | 4.57E+02 | 4.46E+02 | 4 |

| **B** | **Adjusted P Values** | | | | | | | | |
| --- | --- | --- | --- | --- | --- | --- | --- | --- | --- |
| **Treatments** | **FOXG1** | **EMX2** | **KROX20** | **ASCL1** | **DLX2** | **GSX2** | **LHX6** | **LHX8** | **NKX2.1** |
| **Ctr vs. Pu 0.5 µM d1 - d20** | 0.9989 | > 0.9999 | > 0.9999 | > 0.9999 | > 0.9999 | > 0.9999 | > 0.9999 | 0.0003 | 0.0004 |
| **Ctr vs. Pu 0.5 µM d5 - d20** | > 0.9999 | > 0.9999 | > 0.9999 | > 0.9999 | > 0.9999 | > 0.9999 | > 0.9999 | 0.9938 | 0.9218 |
| **Ctr vs. Pu 0.5 µM d10 - d20** | > 0.9999 | > 0.9999 | > 0.9999 | > 0.9999 | > 0.9999 | > 0.9999 | > 0.9999 | 0.9969 | 0.9928 |
| **Pu 0.5 µM d1 - d20 vs. Pu 0.5 µM d5 - d20** | 0.9992 | > 0.9999 | > 0.9999 | > 0.9999 | > 0.9999 | > 0.9999 | > 0.9999 | 0.0007 | 0.0038 |
| **Pu 0.5 µM d1 - d20 vs. Pu 0.5 µM d10 - d20** | 0.9993 | > 0.9999 | > 0.9999 | > 0.9999 | > 0.9999 | > 0.9999 | > 0.9999 | 0.0006 | 0.0011 |
| **Pu 0.5 µM d5 - d20 vs. Pu 0.5 µM d10 - d20** | > 0.9999 | > 0.9999 | > 0.9999 | > 0.9999 | > 0.9999 | > 0.9999 | > 0.9999 | > 0.9999 | 0.9843 |

## Summarized relative expression levels ΔΔCT of marker genes after unsupervised hierarchical linkage clustering

| **A** | **Regimes** | **Genes** | | | | | | |
| --- | --- | --- | --- | --- | --- | --- | --- | --- |
|  |  | **EMX2** | **KROX20** | **NKX2.1** | **FOXG1** | **GSX2** | **ASCL1** | **DLX2** |
|  | **Ctr** | 1.00E+00 | 9.99E-01 | 9.48E-01 | 1.00E+00 | 1.00E+00 | 1.00E+00 | 1.00E+00 |
|  | **SHH 200 ng d5 - d20** | 6.42E-01 | 6.31E-01 | 8.70E+03 | 2.58E+00 | 5.13E+00 | 2.14E+00 | 7.59E+00 |
|  | **SHH 1000 ng d5 - d20** | 4.67E-01 | 8.37E-01 | 4.37E+05 | 2.54E+00 | 4.23E+00 | 2.55E+00 | 7.42E+00 |
|  | **Pu 0.5 µM d5 - d20** | 2.21E-01 | 4.86E-01 | 1.09E+06 | 3.59E+00 | 1.98E+00 | 2.07E+00 | 6.04E+00 |
|  | **Pu 1.5 µM d5 - d20** | 1.26E-01 | 6.40E-01 | 1.15E+06 | 2.78E+00 | 1.32E+00 | 2.70E+00 | 6.50E+00 |

| **B** | **Regimes** | **Genes** | | | | | | | | |
| --- | --- | --- | --- | --- | --- | --- | --- | --- | --- | --- |
|  |  | **ASCL1** | **FOXG1** | **LHX8** | **NKX2.1** | **DLX2** | **LHX6** | **EMX2** | **GSX2** | **KROX20** |
|  | **Ctr** | 1.00E+00 | 1.00E+00 | 9.99E-01 | 1.00E+00 | 1.00E+00 | 9.99E-01 | 1.00E+00 | 1.00E+00 | 1.00E+00 |
|  | **Pu 0.5 µM d5 - d20** | 9.04E-01 | 2.72E+01 | 4.33E+02 | 1.05E+03 | 1.08E+01 | 1.96E+01 | 6.51E-01 | 2.58E+01 | 1.86E-01 |
|  | **Pu 0.5 µM d10 - d20** | 8.19E-01 | 4.05E+01 | 3.45E+02 | 4.57E+02 | 8.18E+00 | 3.72E+00 | 1.21E+00 | 2.34E+01 | 2.98E-01 |
|  | **Pu 0.5 µM d1 - d20** | 1.35E+00 | 2.46E+02 | 7.06E+03 | 6.87E+03 | 1.34E+01 | 3.05E+01 | 9.65E-02 | 3.82E+00 | 1.20E-01 |

## Summary of assessed electrophysiological parameters from hiPSC-derived cholinergic neuron

| **Electrophysiological parameters** | |
| --- | --- |
| Peak Na+-current | -2344 ± 243.7 pA |
| Peak Na+-current/pF | -128.5 ± 13.67 pA/pF |
| Peak K+-current | 2197 ± 205.1 pA |
| Peak K+-current/pF | 132.5 ± 14.81 pA/pF |
| Resting membrane potential | -29.2 ± 1.4 mV |
| Membrane capacitance | 22.5 ± 2.2 pF |
| Input resistance | 748.7 ± 148.2 MΩ |
| Cells with single APs | 36 (65.5%) |
| Cells with train APs | 14 (25.5%) |
| AP amplitude | 57.6 ± 2 mV |
| AP duration | 1.9 ± 0.2 ms |
| AHP amplitude | 6.6 ± 1.9 mV |
| Time to peak AHP | 21 ± 1.3 ms |
| Frequency and percentage of cells with spontaneous large inward currents (>100pA) | 0.15 ± 0.06 Hz (18.2%) |
| Spontaneous large inward current amplitude | 159.1 ± 15.14 pA |
| Frequency and percentage of cells with spontaneous action potentials (>20mV) | 1.74 ± 0.48 Hz (43.6%) |
| Spontaneous action potential amplitude | 45.8 ± 1.3 mV |
| Frequency and percentage of cells with spontaneous miniature postsynaptic currents (>10pA. <100 pA) | 0.51 ± 0.09 Hz (32.7%) |
| Spontaneous miniature postsynaptic current amplitude | 33.6 ± 1.39 pA |

# Supplementary Figures

##

**Supplementary Figure 1.** Relative gene expression (∆CT) per hiPSC line. Data were used for Figure 4B and C.

**Supplementary Figure 2.** Relative gene expression (∆∆CT) per hiPSC line. Data were used for Figure 4B and C.
